# Thieno[2,3-*b*]Pyridine Derivative Targets Epithelial, Mesenchymal and Hybrid CD15s^+^ Breast Cancer Cells

**DOI:** 10.3390/medicines8070032

**Published:** 2021-06-22

**Authors:** Sandra Marijan, Angela Mastelić, Anita Markotić, Nikolina Režić-Mužinić, Nikolina Vučenović, David Barker, Lisa I. Pilkington, Jóhannes Reynisson, Vedrana Čikeš Čulić

**Affiliations:** 1 Department of Medical Chemistry and Biochemistry, University of Split School of Medicine, 21000 Split, Croatia; sandra.dujic.bilusic@mefst.hr (S.M.); angela.mastelic@mefst.hr (A.M.); anita.markotic@mefst.hr (A.M.); nikolina.rezic@mefst.hr (N.R.-M.); nikolina.vucenovic@gmail.com (N.V.); 2 School of Chemical Sciences, The University of Auckland, Auckland 1010, New Zealand; d.barker@auckland.ac.nz (D.B.); lisa.pilkington@auckland.ac.nz (L.I.P.); 3 MacDiarmid Institute for Advanced Materials and Nanotechnology, Wellington 6140, New Zealand; 4 School of Pharmacy and Bioengineering, Keele University, Staffordshire ST5 5BG, UK; j.reynisson@keele.ac.uk

**Keywords:** triple-negative breast cancer, thieno[2,3-*b*]pyridine, CD15s glycosphingolipid, CD15s glycoprotein, CD44, CD24

## Abstract

The adhesion of cancer cells to vascular endothelium is a critical process in hematogenous metastasis and might be similar to the recruitment of leukocytes at the site of inflammation. It is mediated by E-selectin and its ligands, of which the most stereospecific is a glycoconjugate sialyl Lewis x (CD15s), which may be expressed as an oligosaccharide branch of the CD44 glycoprotein, as well as a self-contained glycosphingolipid. It is also known that increased sialylation of glycoconjugates is a feature of malignant cells. The aim of the study was to analyse the effect of a novel thieno[2,3-*b*]pyridine, compound **1**, in MDA-MB-231 triple-negative breast cancer cells (TNBCs) upon CD15s and CD44 expression in different cell subpopulations using flow cytometry. CD15s expression was compared between mesenchymal-like cancer stem cells (CSC, CD44^+^CD24^−^), epithelial cells without CD44 (CD44^−^CD24^+^ and CD44^−^CD24^−^), and CD44^+^CD24^+^ cells that exhibit mesenchymal and epithelial features. In addition, expression of CD44 in CD15s^+^CSC and CD15s^−^CSC was determined. Compound **1** significantly decreased the percentage of CD15s^+^CSC, CD15s^+^CD44^+^CD24^+^, and CD15s^+^CD44^−^ subpopulations, as well as the expression of CD15s in CD44^+^CD24^+^ and CD44^−^ cells, and therefore shows potential as a treatment for TNBC.

## 1. Introduction

The adhesion of cancer cells to vascular endothelium is critical to hematogenous metastasis. It is hypothesised that the shear-resistant adhesion of cancer cells is similar to leukocyte recruitment at the site of inflammation, which is known to be mediated by endothelial (E) selectin and its ligands [1]. Due to E-selectin expression on bone marrow microvascular endothelium [2], it is frequently the site of cancer metastasis [3]. The most stereospecific E-selectin ligand is a glycoconjugate sialyl Lewis x (CD15s). Increased sialylation of glycoconjugates is a feature of malignant cells [4]. Characterisation of glycans expressed in breast cancers, using a panel of antibodies, revealed that BT-20 cells express CD15s as a glycoprotein, while glycosphingolipid CD15s is a functional E-selectin ligand of MDA-MB-468 cells [5]. In the case of CD15s glycoprotein, [NeuAc-Gal-(Fuc)-GlcNAc] terminal tetrasaccharide is linked to the CD44 transmembrane glycoprotein [6]. CD44 variant isoform 4 (CD44v4) is a major glycoprotein decorated with CD15s moieties in metastatic MDA-MB-231 cells [7], whilst more recent research from Shirure et al., using protease treatment, proved that glycosphingolipid CD15s in breast cancer cells is the E-selectin ligand, and there is the potential this could be targeted to prevent metastasis [5].

A critical factor in the malignant breast cancer state is intratumoural cellular heterogeneity, which is also a factor for persistent tumour growth, metastasis, and therapeutic resistance [8]. Conventional chemotherapy preferentially targets proliferating cells which can leave behind resistant stem-like cells, which can cause recurrence [9]. Within the TNBC cell lines BT-549 and Hs 578T, there are three different subpopulations [10]: (1) the CD44^+^CD24^−^ cells characterise the breast cancer stem mesenchymal-like phenotype (cancer stem cells, CSC), (2) the CD44^−^CD24^+^ cells are associated with epithelial cells and are largely present in distant metastases [11], and (3) the CD44^+^CD24^+^ cells with mesenchymal and epithelial features are plastic and able to self-renew [12]. Due to the different signalling pathways in epithelial and mesenchymal cancer stem populations, they respond differently to treatments. Dual inhibition of epithelial and mesenchymal CSC would therefore seem essential for the effective treatment of TNBC [13].

The family of anticancer thieno[2,3-*b*]pyridines was discovered by virtual screening against the phosphoinositide specific-phospholipase C-γ2 target, and it was soon established that they were effective against many different cancer cell lines [14]. This is due to the many cancer-related biological targets that the thieno[2,3-*b*]pyridines modulate [15].

**Figure 1:**
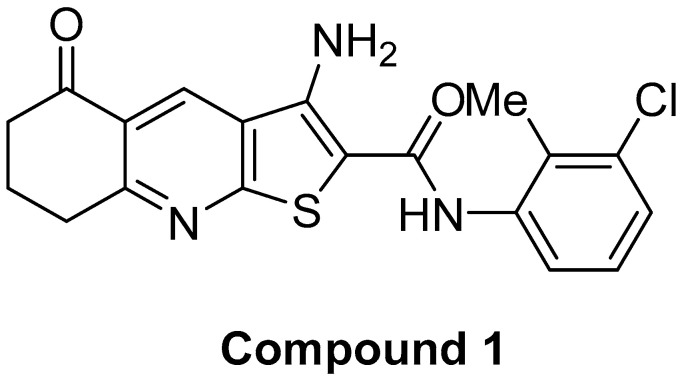
The molecular structure of the anticancer compound **1**, the anticancer thieno[2,3-*b*]pyridine derivative.

Recently, we have described glycosphingolipid expression in breast cancer stem cells after novel thieno[2,3-*b*]pyridine anticancer compound treatment [16] with six gangliosides (GM3, GD3, GM2, GalNacGM1b, IV^3^Neu5Ac-nLc_4_Cer, and IV^6^Neu5Ac-nLc_4_Cer) and three neutral GSLs (Gg_3_Cer, Gb_4_Cer, and nLc_4_Cer) being examined. In this study, we analysed the effect of the same compound treatment in MDA-MB-231 TNBC upon CD15s and CD44 expression in different breast cancer subpopulations. Recently, performing the 3-(4,5-dimethylthiazolyl-2)-2,5-diphenyltetrazolium bromide (MTT) assay, we obtained 60% survived MDA-MB-231 cells after treatment with 2 µM of compound **1** (Figure 1) during 48 h [16].

Therefore, in this study, we used 2 µM of compound **1** and expected that it would not kill all of the cells and be effective upon CD15s expression. A 48 h treatment period was chosen since it is a long enough time to influence cell processes but short enough to yield a sufficient number of survived cells [17]. CD15s expression was compared among mesenchymal-like cancer stem cells (CSC, CD44^+^CD24^−^), epithelial cells without CD44 (CD44^−^CD24^+^ and CD44^−^CD24^−^), and CD44^+^CD24^+^ cells that exhibit mesenchymal and epithelial features. In addition, the expression of CD44 in CSC CD15s^+^ and CD15s^−^ cells was determined.

## 2. Results

**Scheme 1:**
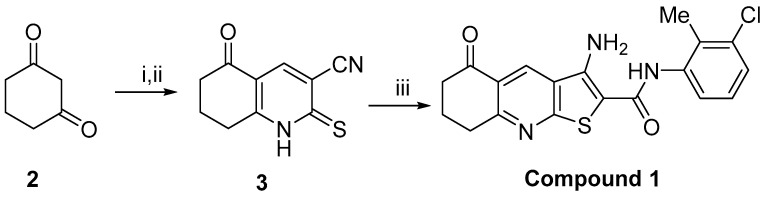
Synthesis of compound **1**. Reagents and conditions: (i) dimethylformamide dimethyl acetal, DMF, 24 h; (ii) cyanothioacetamide, NaH, dimethylformamide, 24 h, 82% (over 2 steps); (iii) 2-bromo-*N*-(3-chloro-2-methylphenyl)acetamide, sodium carbonate, ethanol, 24 h, 28%.

Compound **1** was prepared in three steps from 1,3-cyclohexanedione **2** using our previously reported method (Scheme 1) [18,19,20]. With the aim of ascertaining whether the cytotoxic effect of compound **1** had an impact on the expression of CD15s in CSC (defined as CD44^+^CD24^−^ subpopulation), CD44^+^CD24^+^, and CD44^−^ (defined as CD44^−^CD24^+^ and CD44^−^CD24^−^ subpopulation), we compared the percentages and number of events (a number that corresponds to the number of cells acquired and detected by flow cytometer) within each subpopulation after treatment with compound **1** in relation to untreated cells.

Initially, the expressions of CD15s and CD44 were studied. Expression of CD15s and CD44 per cell is represented with geometric mean fluorescence intensity (GMI) of fluorochromes eFluor 660 and FITC, respectively. The percentage of the cells that are CD15s^+^ is an interesting parameter but has less impact when compared to GMI. In the case of CD15s, GMI reflects the quantity and/or activity of different glycosyltransferases involved in CD15s synthesis.

**Figure 2:**
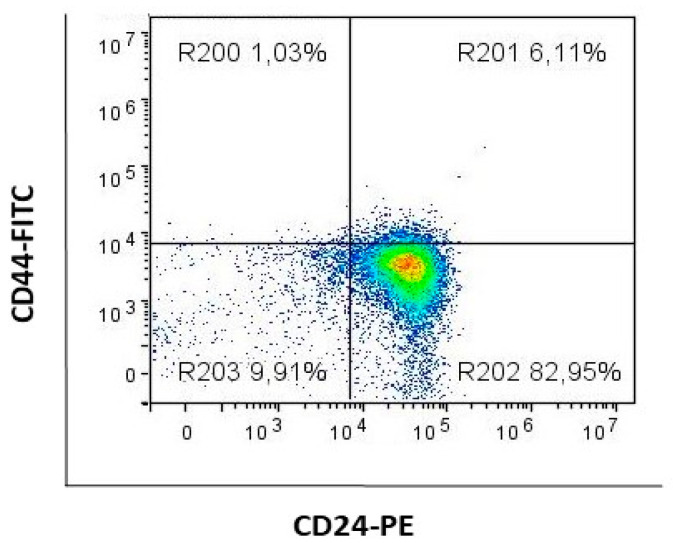
Gating strategy. Notes: representative dot plot of gating CD44^+^CD24^−^ (gate R200), CD44^+^CD24^+^ (gate R201), CD44^−^ (CD44^−^CD24^+^ and CD44^−^CD24^−^, gates R202 and R203, respectively) subpopulations.

Different subpopulations, concerning their CD44 and CD24 expression, were gated as shown in Figure 2.

### 2.1. Compound ***1*** Decreased the Percentage and Number of Events of CD15s^+^ CSC

**Figure 3:**
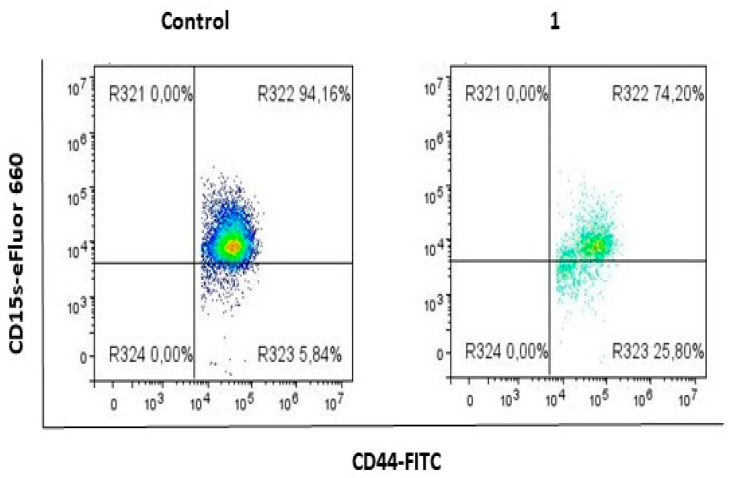
Expression of CD15s in CSC subpopulation after treatment with compound **1**. Notes: R321- CD15^+^CD44^−^ cells; R322- CD15s^+^CD44^+^ cells; R323- CD15s^−^CD44^+^ cells; R324- CD15s^−^CD44^−^. Abbreviations: CSC, cancer stem cells.

A statistically significant decrease (*p*-value < 0.01) in the percentage of CD15s^+^ CSC was obtained upon treatment with compound **1**, whilst the number of CD15s^+^ events was tenfold lower (*p*-value < 0.001) in treated CSC, compared to untreated ones (Figure 3, Table 1).

### 2.2. Compound ***1*** Decreased the Percentage and Number of Events of CD15s^+^CD44^−^ Subpopulation

**Figure 4:**
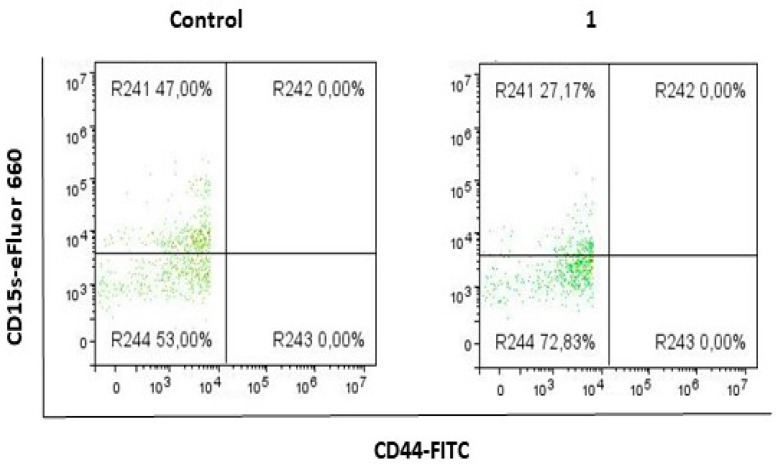
Expression of CD15s in CD44^−^ subpopulation after treatment with compound **1**. Notes: R241- CD15^+^CD44^−^ cells; R242- CD15s^+^CD44^+^ cells; R243- CD15s^−^CD44^+^ cells; R244- CD15s^−^CD44^−^.

Treatment with compound **1** resulted in a decrease in the percentage and events of CD15s^+^ cells of CD44^−^ subpopulation (Figure 4, Table 2). The percentage of CD15s^+^CD44^−^ in treated cells was twice lower when compared to untreated controls (*p*-value < 0.01). The number of CD15s^+^CD44^−^ cells was almost threefold lower in treated cells, compared to untreated controls (*p*-value < 0.01).

### 2.3. Compound ***1*** Decreased the Percentage and Number of Events of CD15s^+^CD44^+^CD24^+^ Subpopulation

**Figure 5:**
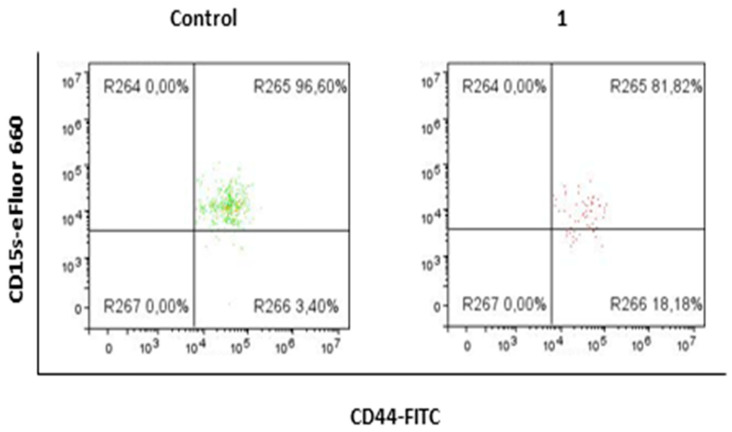
Expression of CD15s in CD44^+^CD24^+^ subpopulation after treatment with compound **1**. Notes: R264- CD15^+^CD44^−^ cells; R265- CD15s^+^CD44^+^ cells; R266- CD15s^−^CD44^+^ cells; R267- CD15s^−^CD44^−^.

After treatment with compound **1**, the percentage and events of CD15s^+^ cells of CD44^+^CD24^+^ subpopulation were also significantly decreased (Figure 5, Table 3). The percentage of CD15s^+^CD44^+^CD24^+^ treated cells was significantly lower (*p*-value < 0.01), compared to untreated controls. The number of CD15s^+^CD44^+^CD24^+^ cells was more than fivefold lower in treated cells, compared to untreated controls (*p*-value < 0.05).

### 2.4. Compound ***1*** Increased the Percentage of CD15^−^ Cells in CSC, CD44^−^ and CD44^+^CD24^+^ Subpopulations

There was no difference in the number of events of CD15s^−^ in CSCs, CD44^−^ and CD44^+^CD24^+^ subpopulations after treatment with compound **1**, whilst this treatment resulted in a several-fold increase in the percentage of CD15s^−^ CSC and CD44^+^CD24^+^, and a significant increase in CD44^−^ subpopulation (*p*-value < 0.01 for all subpopulations) (Table 4). The sum of CD15s^+^ and CD15s^−^ percentages in each sample is equal to 100%, as proved by adding percentages from Table 1, Table 2, Table 3 and Table 4.

### 2.5. Geometric Mean Fluorescence Intensity (GMI)

**Figure 6:**
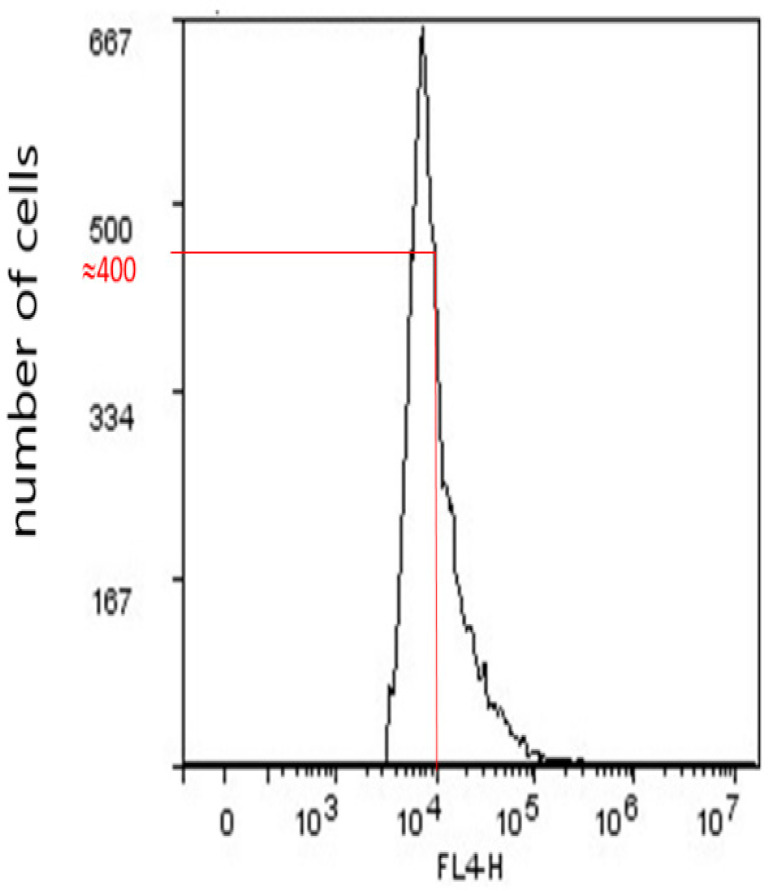
Strategy of GMI calculation. Notes: Approximately 400 cells have a fluorescence intensity of 10,000.

Geometric mean fluorescence intensity (GMI), for instance, of CD15s (CD15s expression/cell), was derived in FlowLogic from a number of cells vs. log of fluorescence in FL4 flow cytometer channel, with a standard optical filter of 675/25 nm (Figure 6). The emission wavelength of immunofluorescence of eFluor 660 fluorochrome that is conjugated to a secondary anti-CD15s antibody is 660 nm.

GMI is derived by multiplying cell numbers with corresponding fluorescences, adding all results, and dividing the total sum with the total cell number.

### 2.6. Expression of CD15s in CSC, CD44^−^, and CD44^+^CD24^+^ Subpopulations after Treatment with Compound ***1***

**Figure 7:**
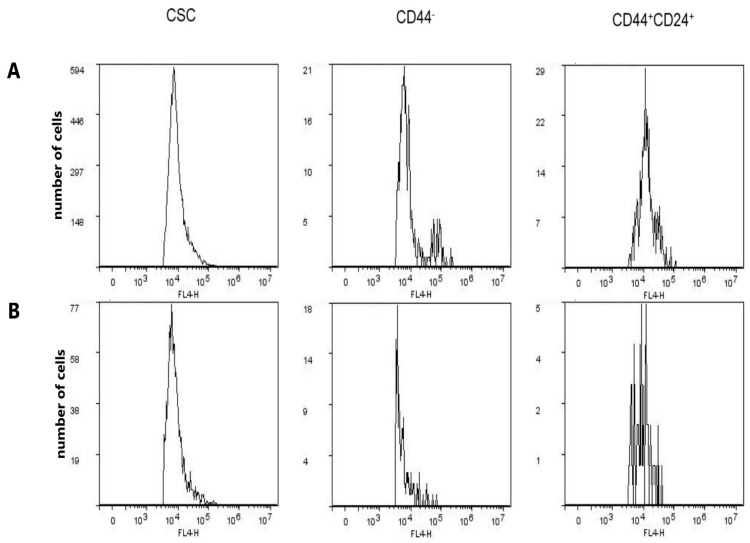
Immunofluorescences of secondary antibody to anti-CD15s in control (**A**) and compound **1** treated cells (**B**). Notes: secondary antibody was conjugated with eFluor 660 fluorochrome. Fluorescences were measured in FL4 channel of flow cytometer for CD15s^+^ CSC, CD15s^+^CD44^−^, and CD15s^+^CD44^+^CD24^+^ cells. Abbreviations: CSC, cancer stem cells.

There was no difference in the expression of CD15s in CSCs after treatment with compound **1**, whilst this treatment resulted in a significant decrease (*p*-value < 0.01) in CD15s expression in CD44^−^ and CD44^+^CD24^+^ subpopulations (Figure 7, Table 5).

### 2.7. Expression of CD15s in Untreated CSC and CD44^+^CD24^+^ Cells

**Figure 8:**
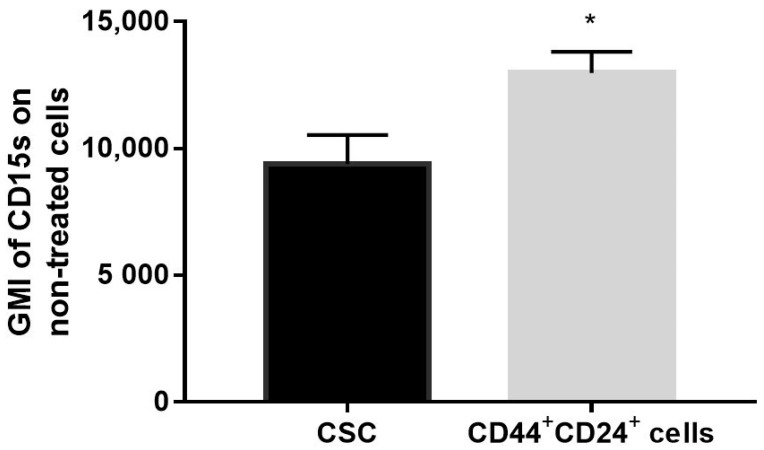
Expression of CD15s in untreated CSC and CD44^+^CD24^+^ subpopulation. Notes: geometric mean fluorescence intensity of CD15s in CD15s^+^CSC and CD15s^+^CD44^+^CD24^+^ cells without treatment with compound **1**. Data are expressed as a mean from experiment performed in triplicate ± SD. Columns, mean of cells; bars, SD; * *p* < 0.05. Abbreviations: CSC, cancer stem cells; GMI, geometric mean fluorescence intensity.

Geometric mean fluorescence intensity (GMI) of CD15s (CD15s expression/cell) was increased in untreated CD44^+^CD24^+^ subpopulation (*p*-value < 0.05), compared to untreated CSC (Figure 8).

### 2.8. Expression of CD44 in CSC Positive and Negative for CD15s after Treatment with Compound ***1***

**Figure 9:**
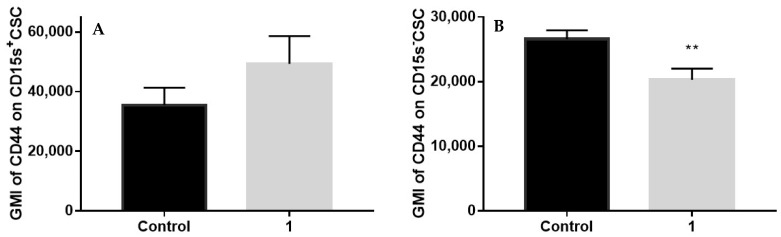
Expression of CD44 in CSC positive and negative for CD15s after treatment with compound **1**. Notes: geometric mean fluorescence intensity of CD44 in CD15s^+^CSC (**A**) and CD44 in CD15s^−^CSC (**B**) after treatment with compound **1**. Data are expressed as a mean from experiment performed in triplicate ± SD. Columns, mean of cells; bars, SD; ** *p* < 0.01. Abbreviations: CSC, cancer stem cells; GMI, geometric mean fluorescence intensity.

Treatment with compound **1** did not influence CD44 expression in CD15s^+^CSC (Figure 7A) but decreased expression (*p*-value < 0.01) of CD44 in CD15s^−^CSC (Figure 9).

## 3. Discussion

Our previous investigation demonstrated the efficiency of 3-amino-*N*-(3-chloro-2-methylphenyl)-5-oxo-5,6,7,8-tetrahydrothieno[2,3-*b*]quinoline-2-carboxamide (compound **1**) in lowering the CSCs in two TNBC lines, MDA-MB-231 and MDA-453, characterised as CD44+CD24- and CD133+, respectively [16]. We have also shown that cell migration and morphology of the MDA-MB-231 cell line are significantly affected by treatment with compound **1** [21]. In this study, we analysed adhesion molecules CD15s and CD44 in different TNBC subpopulations. We have found that compound **1** significantly decreased the number and percentage of CD15s^+^ cells within three subpopulations: CD44^+^CD24^−^ cancer stem cells, CD44^−^ epithelial cells, and CD44^+^CD24^+^ cells that exhibit mesenchymal and epithelial features (Figure 3, Figure 4 and Figure 5). These three subpopulations are different concerning their CD15s plasma membrane anchoring. Due to their CD44 content, CD44^+^CD24^−^ and CD44^+^CD24^+^ cells are expected to express CD15s as glycoproteins [7], while the expression of CD15s found in CD44^−^ cells could be attributed to glycosphingolipid CD15s [5].

The presence of CD15s as a branch of CD44 glycoprotein was proved as a selectin ligand activity of CD44. Although *O*-glycosylated, sialofucosylated CD44v isoforms are the prevalent E-selectin ligands expressed by colon carcinoma cells, it has been shown that in hematopoietic cells, selectin ligand activity of CD44 is primarily associated with the *N*-glycosylated, sialofucosylated CD44s isoform [22,23]. Quantitative RT-PCR (qRT-PCR) using primers designed for the specific isoforms allowed a comparison of the expression of CD44 isoforms at the mRNA level [24]. At mesenchymal-like MDA-MB-231 cells, CD44s, showing limited E-selectin ligand activity, was described as dominant, in comparison to CD44v [25], which is expressed in the epithelial-like BT-20 breast cancer cell line. During metastasis, tumour cells undergo epithelial-to-mesenchymal transition [26], as well as the reverse, mesenchymal-to-epithelial transition [27]. The mesenchymal state is beneficial for cell invasion in tissue [28], and functional E-selectin ligands are not necessary for these processes. On the other hand, the epithelial state, with E-selectin ligands, may be required for the adhesion of circulating tumour cells to vascular endothelium. In our study, there was no difference in CD44 expression between untreated CSC and CD44^+^CD24^+^ (data not shown), while the expression of E-selectin ligand CD15s in untreated CD44^+^CD24^+^ cells that exhibit mesenchymal and epithelial features was higher, in comparison to untreated mesenchymal CSC (Figure 8), emphasising the role of CD44^+^CD24^+^ in vascular endothelium adhesion.

We found that upon treatment with compound **1**, there was a reduction in the CD15^+^ CSC subpopulation percentage and number (Figure 3) but without influence upon CD15s and CD44 expression in CD15^+^ CSC (Figure 7 and Figure 9A). Compound **1** decreased expression of CD44 in CD15^−^ CSC cells, while the increase of mean GMI of CD44 in CSC CD15^+^ cells was not statistically significant.

Resistance of CD44 expression in CD15s^+^CSC could be a consequence of their more resistant endoplasmic reticulum to compound **1** treatment, in comparison to CD15^−^CSC. Namely, compound **1** could induce endoplasmic reticulum stress, as described for thienopyridine clopidogrel gastric epithelial cells [29]. The endoplasmic reticulum is an organelle in which the final CD44 glycoprotein synthesis is located [30]. We can hypothesise that in CSC CD15s^+^ cells, the endoplasmic reticulum is preserved.

Compound **1** did not affect CSC CD15s, but CD15s expression decline was detected in subpopulations without CD44, as well as at CD44^+^CD24^+^ cells (Figure 7). We have shown that compound **1** lowers CSC, CD44^+^CD24^+,^ and CD44^−^ subpopulations and expression of CD15s in CD44^+^CD24^+^ and CD44^−^ cells, showing that it or similar analogs could be developed into new treatments for triple-negative breast cancer.

Thieno[2,3-*b*]pyridines are known to modulate the activity of numerous molecular targets, including G protein-coupled receptors [15], P2Y12 receptors [31], tyrosyl DNA phosphodiesterase 1–a DNA repair enzyme [32], the colchicine binding site of tubulin [33], phospholipase C-δ1 [21] as well as PIM-1 [34] and eukaryotic elongation factor-2 kinase [35], and cyclooxygenases [36]. It can therefore be stated that compound **1** is modulating many processes affecting E-selectin.

In order to to reduce metastasis in vivo, it may be necessary to target the E-selectin ligand activity of CD44. It is possible that knockdown of CD44v in breast cancer cells may increase their metastatic potential due to possible facilitated epithelial-to-mesenchymal transition [37,38]. Glycosphingolipid CD15s E-selectin ligands, expressed in circulating tumour cells are less sensitive to epithelial-to-mesenchymal transition regulation, being able to serve as the critical adhesion molecules, which mediate adhesion to vascular walls [5,25].

An ideal therapeutic substance should target both heterogeneous epithelial and mesenchymal cancer cells, including CD44^−^/CD24^−^ cells. Recently, Vikram et al. showed that high tumourigenicity in MDA-MB-231 cells is not CD44 dependent [39]. CD44^+^CD24^−^ and CD44^−^/CD24^−^ populations have comparable metastatic efficiency. CD44^−^ population forms slightly larger tumours, and mice injected with this population show higher mortality. We have shown that compound **1** lowers CSC, CD44^+^CD24^+^, and CD44^−^ subpopulations and expression of CD15s in CD44^+^CD24^+^ and CD44^−^ cells, showing that it or similar analogues could be developed into new treatments for triple-negative breast cancer. It has been shown that epithelial and mesenchymal CSC are spatially separated in vivo breast cancer tumours [40,41] and that the tumour microenvironment could be essential for tumour survival. Further in vivo experiments, investigating glycosidase and glycosyltransferase activity, are required to define the exact mechanisms induced by compound **1** and its efficiency in animal TNBC models.

## 4. Materials and Methods

### 4.1. Chemistry and Cell Line

Pure samples of 3-amino-*N*-(3-chloro-2-methylphenyl)-5-oxo-5,6,7,8-tetrahydrothieno[2,3-*b*]quinoline-2-carboxamide (compound **1**) were prepared from 1,3-cyclohexadione **2** using the reported methods [16,18,19,20].

Synthesis of carbonitrile **3**: A solution of cyclohexanedione **2** (0.50 g, 4.46 mmol) and DMF-DMA (0.59 mL, 4.46 mmol) in DMF (15 mL) was stirred for 24 h at room temperature. To the above solution a pre-prepared mixture of sodium hydride (0.21 g, 8.92 mmol) and cyanothioacetamide (0.45 g, 4.46 mmol) in DMF (15 mL), which had been mixed for 10 min, was then added and the entire mixture stirred for 24 h. The mixture was then acidified to pH 4 using 10M HCl and stirred for 24 h. The resultant solid was filtered and recrystallised using ethanol to give the carbonitrile **3** (0.73 g, 82%) as a brown solid. m.p. >350 °C. [lit. m.p. >300 °C]; ^1^H NMR (400 MHz; (CD_3_)_2_SO) 2.02–2.08 (2H, m, H-7), 2.51–2.54 (2H, m, H-6), 2.99–3.02 (2H, m, H-8), 8.24 (1H, s, H-4), 14.4 (1H, s, NH); 13C NMR (100 MHz; DMSO-d6) 19.9 (C-8), 26.7 (C-9), 36.5 (C-7), 114.8 (CN) 116.2 and 118.2 (C-5 and C-3), 140.1 (C-4), 161.4 (C-2), 180.6 (C-10), 192.5 (C = O). The ^1^H-NMR data agreed with the literature [42].

Synthesis of 3-amino-*N*-(3’-chloro-2’-methylphenyl)-5-oxo-5,6,7,8-tetrahydrothieno[2,3-*b*]quinoline- 2-carboxamide (compound **1**): A mixture of 2-bromo-*N*-(3-chloro-2-methylphenyl)acetamide (0.13 g, 0.49 mmol), carbonitrile **3** (100 mg, 0.49 mmol) and sodium carbonate (60 mg, 0.57 mmol) in absolute ethanol (2 mL) was heated at reflux for 18 h. After cooling to room temperature, solvent was removed in vacuo and the resultant solid was washed with water. The remaining solid was recrystallised from methanol to give compound **1** (54 mg, 28%) as a yellow solid. m.p. > 230 °C. ν_max_(ATR)/cm^−1^ 3425, 3307, 1672, 1576, 1513, 1427, 1249, 1096; δ_H_ (400 MHz, (CD_3_)_2_SO) 2.15 (2H, p, *J* = 6.0 Hz, H-7), 2.23 (3H, s, CH_3_), 2.72 (2H, t, *J* = 6.0 Hz, H-6), 3.19 (2H, t, *J* = 6.0 Hz, H-8), 7.21–7.28 (2H, m, H-5’ and H-6’), 7.35 (1H, dd, *J* = 7.6, 1.2 Hz, H-4’), 7.50 (2H, br s, NH_2_), 9.03 (1H, s, H-4), 9.46 (1H, br s, NH). δ_C_ (100 MHz, (CD_3_)_2_SO) 15.5 (CH_3_), 21.2 (C-7), 32.4 (C-8), 38.1 (C-6), 96.0 ( C-2), 124.4 (C-4a), 125.2 (C-3a), 126.2 (C-6’), 126.6 (C-5’), 126.7 (C-4’), 130.1 (C-4), 132.6 (C-2’), 133.6 (C-1’), 138.1 (C-3’), 162.2 (C-9a), 163.9 (C-8a), 164.0 (C = O), 197.1 (C-5); *m*/*z* (ESI^+^): 410 (^37^ClMNa^+^, 39%), 408 (^35^ClMNa^+^, 100%); HRMS (ESI^+^) found (^37^ClMNa^+^): 410.0550, C_19_H_16_^37^ClN_3_NaO_2_S requires 410.0519. Found (^35^ClMNa^+^): 408.0558, C_19_H_16_^35^ClN_3_NaO_2_S requires 408.0544.

Just prior to its use in this study, compound **1** was dissolved in DMSO. Cell line MDA-MB-231 was grown in a humidified incubator at 37 °C and 5% CO_2_ in Dulbecco’s modified Eagle medium (DMEM, Sigma-Aldrich, Steinheim, Germany) containing 10% fetal bovine serum (EuroClone, Milan, Italy) and 1% antibiotics (EuroClone).

### 4.2. Flow Cytometric Analysis

An equal number of cells were seeded in six-well plates and treated with 2 μM compound **1** for 48 h, as well as the untreated controls, trypsinised and washed with phosphate-buffered saline (PBS). Cells were pre-treated with an Fc receptor blocking reagent (Miltenyi Biotec GmbH, Bergisch Gladbach, Germany) to prevent nonspecific binding. After 15 min of incubation with PBS-diluted anti-CD15s (1:3; BD Biosciences, Inc., San Diego, CA, USA) at room temperature, cells were incubated with diluted anti-CD44-FITC (1:13; BD Biosciences), anti-CD24-PE (1:3; eBioscience), and secondary antibody to anti-CD15s conjugated with eFluor 660 fluorochrome (1:10; eBioscience) for 15 min in the dark, resuspended in PBS, and thereafter analysed by flow cytometry. Data acquisition of triple-stained samples was performed on a BD Accuri C6 cytometer (BD Biosciences) and analysed using the FlowLogic Software.

### 4.3. Statistical Analysis

For statistical analyses, student t-test was performed using statistical software GraphPad Prism 7.0 (San Diego, CA, USA) with the significance set at *p* < 0.05.

## Figures and Tables

**Table 1:** Percentage and events of CD15s in CSC after treatment with compound **1**.

	CSC		
	Control	1	*p*-Value
% CD15s^+^	92.83 ± 3.21	74.15 ± 2.21	0.0012
Events CD15s^+^	11,585 ± 977	1263 ± 107	0.00005

Notes: Data are expressed as a mean from experiment performed in triplicate ± SD. Abbreviations: CSC, cancer stem cells.

**Table 2:** Percentage and events of CD15s in CD44^−^ subpopulation after treatment with compound **1.**

	CD44^−^		
	Control	1	*p*-Value
% CD15s^+^	49.03 ± 5.74	27.06 ± 5.75	0.0094
Events CD15s^+^	429 ± 70	153 ± 20	0.0028

Notes: Data are expressed as a mean from experiment performed in triplicate ± SD.

**Table 3:** Percentage and events of CD15s in CD44^+^CD24^+^ subpopulation after treatment with compound **1**.

	CD44^+^CD24^+^		
	Control	1	*p*-Value
% CD15s^+^	93.15 ± 3.64	82.77 ± 0.82	0.0052
Events CD15s^+^	370 ± 176	69 ± 8	0.0943

Notes: Data are expressed as a mean from experiment performed in triplicate ± SD.

**Table 4:** Percentage and events of CD15s^−^ cells in CSC, CD44^−^ and CD44^+^CD24^+^ subpopulations after treatment with compound **1.**

	CSC			CD44^−^			CD44^+^CD24^+^		
	Control	1	*p*-Value	Control	1	*p*-Value	Control	1	*p*-Value
% CD15s^−^	7.17 ± 3.21	25.85 ± 2.21	0.0019	50.97 ± 5.74	72.94 ± 5.75	0.0094	4.36 ± 2.66	16.4 ± 1.56	0.0052
Events CD15s^−^	668 ± 33.94	443 ± 75.44	0.1758	444.67 ± 50.29	426.33 ± 105.67	0.8045	14 ± 2	13.67 ± 2.51	0.8666

Notes: Data are expressed as a mean from experiment performed in triplicate ± SD. Abbreviations: CSC, cancer stem cells.

**Table 5:** Expression of CD15s in CSC, CD44^−^ and CD44^+^CD24^+^ subpopulations after treatment with compound **1.**

	CSC			CD44^−^			CD44^+^CD24^+^		
	Control	1	*p*-Value	Control	1	*p*-Value	Control	1	*p*-Value
GMI of CD15s	9396 ± 1138	9212 ± 1006	0.844	9220 ± 899	6090 ± 191	0.0041	12985 ± 835	9469 ± 592	0.004

Notes: Data are expressed as a mean from experiment performed in triplicate ± SD. Abbreviations: CSC, cancer stem cells; GMI, geometric mean fluorescence intensity.

## Data Availability

The data presented in this study are available on request from the corresponding author.
